# Infrared-emitting, peptidase-resistant fluorescent ligands of the bradykinin B_2_ receptor: application to cytofluorometry and imaging

**DOI:** 10.1186/s13104-016-2258-1

**Published:** 2016-09-26

**Authors:** Lajos Gera, Xavier Charest-Morin, Melissa Jean, Hélène Bachelard, François Marceau

**Affiliations:** 1 Department of Biochemistry, University of Colorado Denver, Aurora, CO 80045 USA; 2 Axe maladies infectieuses et immunitaires, Centre de recherche, CHU de Québec Université Laval, Quebec, QC G1V 4G2 Canada; 3 Axe endocrinologie et néphrologie, Centre de recherche, CHU de Québec Université Laval, Quebec, QC G1V 4G2 Canada; 4 Centre de Recherche du CHU de Québec (CHUL), Room T1-49, 2705 Boulevard Laurier, Quebec City, QC G1V 4G2 Canada

**Keywords:** Bradykinin B_2_ receptors, Fluorescence, Cyanine dye 7, Human umbilical vein, Microscopy, Cytofluorometry

## Abstract

**Background:**

We have previously reported the design, pharmacological properties and imaging application of bradykinin (BK) B_2_ receptor (B_2_R) ligands conjugated with fluorophores such as fluorescein derivatives at their N-terminus. To take advantage of the high penetration of infrared light into living tissues and their low autofluorescence in this region of the spectrum, additional probes conjugated with cyanine dye 7 (Cy7) were synthesized and characterized.

**Results:**

The antagonist B-9430 (D-Arg-[Hyp^3^,Igl^5^,D-Igl^7^,Oic^8^]-BK) and the agonist B-9972 (D-Arg-[Hyp^3^,Igl^5^,Oic^7^,Igl^8^]-BK) were N-terminally extended with the infrared fluorophore Cy7, producing the peptides B-10665 and B-10666, respectively. Pharmacological studies indicated that the agonist B-10666 lost much affinity for the B_2_R vs. the parent peptide, whereas the antagonist B-10665 better retained its potency vs. B-9430 (competition of [^3^H]BK binding to human B_2_R, contractility of the human isolated umbilical vein for which potency losses were more important in each case). Both probes stained HEK 293 cells that expressed the B_2_R-green fluorescent protein (GFP) construction in a specific manner (confocal microscopy) and with very extensive co-localization of the green and infrared fluorescence in either case. The agonist B-10666 at 100 nM promoted the endocytosis of B_2_R-GFP in live cells, but not the antagonist version at 10–25 nM. The Cy7-labeled peptides did not label cells expressing the β_2_-adrenoceptor-GFP construction. B-10665 at low nanomolar concentrations was an effective probe for the recombinant B_2_Rs in cytofluorometry and macroscopic imaging of cell wells (IVIS imaging system operated for infrared fluorescence detection).

**Conclusions:**

Despite a propensity for non-specific binding when used at high concentrations and limited sensitivity, Cy7-conjugated peptidase-resistant B_2_R ligands support original imaging and cytofluorometric applications.

**Electronic supplementary material:**

The online version of this article (doi:10.1186/s13104-016-2258-1) contains supplementary material, which is available to authorized users.

## Background

We have previously reported the design, pharmacological properties and imaging application of bradykinin (BK) B_2_ receptor (B_2_R) ligands conjugated with fluorophores such as fluorescein derivatives or AlexaFluor-350 at their N-terminus, with application to microscopy in cells that expressed recombinant receptors and their molecular partners such as arrestins, angiotensin converting enzyme and Rab small GTPases [1–4].

To take advantage of the high penetration of infrared light into living tissues and their low autofluorescence in this region of the spectrum, we wished to produce and characterize additional probes conjugated with a suitable fluorophore, cyanine dye 7 (Cy7). The parent peptides for the antagonist and the agonist versions are B-9430 (D-Arg-[Hyp^3^,Igl^5^,D-Igl^7^,Oic^8^]-BK) and B-9972 (D-Arg-[Hyp^3^,Igl^5^,Oic^7^,Igl^8^]-BK), respectively. They are well characterized pharmacologically [5]. The major kinin-inactivating ectopeptidases are angiotensin converting enzyme and aminopeptidase P [6, 7] and both parent peptides are intrinsically resistant to them due to extensive substitutions with non-natural amino acid residues (Table 1). Thus, the Cy7-conjugated versions, termed B-10665 and B-10666, respectively, were produced, pharmacologically characterized and exploited in original imaging experiments.

**Table 1:** Sequences of novel BK analogs with N-terminal extensions compared to that of parent peptides in each category (italicized names)

Peptide	Position											Binding competition IC_50_ nM (95 % C.L.)
	−1	0	1	2	3	4	5	6	7	8	9	
B_2_ receptor agonists												
First parent peptide: BK			Arg	Pro	Pro	Gly	Phe	Ser	Pro	Phe	Arg-COOH	8.2 (6.0–11.2)
Second parent peptide: B-9972		D-Arg	Arg	Pro	Hyp	Gly	Igl	Ser	Oic	Igl	Arg-COOH	17.3 (13.3–22.4)
B-10666 = Cy7-B-9972	Cy7	D-Arg	Arg	Pro	Hyp	Gly	Igl	Ser	Oic	Igl	Arg-COOH	598 (481–742)
B_2_ receptor antagonists												
Parent peptide: B-9430		D-Arg	Arg	Pro	Hyp	Gly	Igl	Ser	D-Igl	Oic	Arg-COOH	42.6 (20.0–90.8)
B-10665 = Cy7-B-9430	Cy7	D-Arg	Arg	Pro	Hyp	Gly	Igl	Ser	D-Igl	Oic	Arg-COOH	25.7 (11.5–57.1)

Cy7, cyanine dye 7; Hyp, *trans*-4-hydroxyproline; Igl, α-(2-indanyl)glycine; Oic, (3as, 7as)-octahydroindole-2-carboxylic acid; Tic, 1,2,3,4-tetrahydroisoquinoline-3-carboxylic acid

## Methods

### Synthesis of Cy7-peptide conjugates

Trifluoroacetate salts of B-9430 and B-9972 (18.0 mg, 0.01 mmol) were mixed respectively in 2.5 mL DMF with Cy7-NHS ester (6.82 mg, 0.01 mmol; CAS: 1432010-64-1; C_41_H_48_N_3_O_4_·Cl; Lumiprobe Corp., Hallandale Beach, FL, USA), HOBt (1.51 mg, 0.01 mmol) and DIEA (13.1 µL; 0.075 mmol) at room temperature for 10 h (Additional file 1: Figure S1). The Cy7-BK conjugates, termed B-10665 and B-10666, respectively, were purified by preparative HPLC on a Vydac C18 column using a gradient of 20–65 % acetonitrile/water containing 0.1 % TFA (Additional file 1: Figure S2, shows an example of a HPLC trace, that of B-10665). Their identities were confirmed by LC–MS (Additional file 1: Figure S3). The solubility of Cy7-conjugated peptides in aqueous media was much inferior to that of the parent peptides, and stock solutions were made in DMSO. The final DMSO concentration in pharmacological experiments was always inferior to 0.1 % (v/v). BK was purchased from Bachem (Torrance, CA, USA) and the B_2_R antagonist icatibant, from Phoenix Pharmaceuticals (Burlingame, CA, USA).

### Radioligand binding assay

The binding of [^3^H]BK ([2,3-prolyl-3,4-^3^H(N)]-bradykinin, 85.4 Ci/mmol, PerkinElmer, Boston, MA, USA) to adherent intact cells expressing a form of the B_2_R was evaluated precisely as described [8]. HEK 293a cells stably expressing myc-tagged human B_2_Rs [8] were used to estimate receptor affinity via the binding competition of a fixed concentration of the radioligand (3 nM) with several unlabeled peptides applied in a wide concentration range.

### Contractility assays

Local ethics review boards approved the anonymous use of segments of human umbilical cords obtained following elective cesarean sections; informed consent was obtained from the mothers. Novel Cy-7 conjugated B_2_R ligands were assayed via the contraction of the human isolated umbilical vein [9, 10]. Cumulative concentration effect curves were constructed for the agonist version and its potency compared to that of BK. The antagonist version was introduced 30 min before the construction of the concentration-effect curves of the reference agonist BK and the shift to the right was used to determine their potencies using the Schild regression and pA_2_ scale [1].

### Cytofluorometry

A subclone of HEK 293 cells, called HEK 293a, originally obtained from Sigma-Aldrich was used in these experiments, either as a non-transfected cell type or as one stably expressing myc-tagged human B_2_Rs [8]. These cells were grown in Dulbecco’s modified Eagle’s medium supplemented with 10 % fetal bovine serum, 1 % l-glutamine, and 1 % penicillin–streptomycin stock solutions (100×). Cells were detached using the protease-free Cell Dissociation Buffer (Invitrogen), incubated in D-MEM without serum at 37 °C for 30 min under agitation in the presence of a Cy7-conjugated ligands and other drugs, rapidly centrifuged (30 s, 11,000*g*) and resuspended in phosphate buffered saline. Then, the fluorescence of the cell suspensions was assessed using the BD SORP LSR II cell analyzer for the uptake of the as a function of stimulation and transgene expression. The cytofluorometry results were analyzed using the BD FACS DIVA software.

### Microscopy

HEK 293 cells, either not transfected or stably expressing a fully functional rabbit B_2_R construction fused to green fluorescent protein (B_2_R-GFP) [2, 11] were grown as described and plated on poly-l-lysine precoated glass surfaces. B_2_R-GFP is a high affinity, fully functional receptor that supports subcellular localization in intact cells; further, the fusion protein is not degraded by short treatments with various ligands (immunoblot evidence) [5] and is not expected to participate to any energy transfer with Cy7-labeled ligands. As a specificity control, other HEK 293 cells were transiently transfected with a vector coding for the β_2_-adrenoceptor-GFP Topaz fusion protein [12] (the vector is a generous gift from Prof. Michel Bouvier, Université de Montréal) using polyethylenimine as described [13]. Cells were generally treated for 30 min with Cy7-conjugated ligands (incubation carried out at 37 °C in humidified atmosphere containing 5 % CO_2_), rinsed 4–5 times with phosphate buffered saline, observed in microscopy for green and infrared epifluorescence and differential interference contrast microscopy (DIC) using a Quorum Wave FX confocal spinning disk microscope coupled to a Hamamatsu ImageEM digital camera (filters for GFP: excitation 491 nm, emission 536 nm; for Cy7: excitation 642 nm, emission 810). The objective was Leica HCX PL APO 63X/1,30 glycerol immersion lens.

### Imaging of infrared fluorescence in macroscopic objects

The integrated imaging station IVIS Lumina LT Series III (PerkinElmer), along with its Living Image software, was exploited to evaluate the distribution of B_2_Rs in macroscopic object using the intrinsic infrared fluorescence of the antagonist B-10665. The apparatus was set in fluorescence mode with filters at 675 nm (excitation) and 831 nm (emission), medium binning and 60 s exposure time. This experiment exploited HEK 293a cells grown in 24-well plates that stably expressed either human or rat B_2_R sequences N-terminally tagged with the myc epitope [8]. Expression of these receptors as a function of pharmacologic treatment was controlled in separate plates of cells that were not fixed or permeabilized using the binding of anti-myc monoclonal antibody (clone 4A6, Millipore, dilution 1:1000) added along goat horseradish peroxidase-conjugated anti-mouse IgG antibodies (Santa Cruz, dilution 1:1000). After 15 min of incubation at 37 °C and 4 washing with phosphate buffered saline, the reaction was revealed by adding the TrueBlue substrate for immunohistochemistry (Kirkegaard & Perry Lab, Inc., Gaithersburg, MD, USA) used as directed. The resulting blue precipitate was photographed at the macroscopic scale.

### Data analysis

Results are presented as mean ± SEM. Radioligand binding data were fitted by nonlinear regression to a one-site competition equation using a least-square method (Prism 5.0, GraphPad Software Inc., San Diego, CA, USA) and IC_50_ values with their 95 % confidence limits were calculated from this procedure. The same computer program was used to draw concentration-effect curves (least square fitting of sigmoidal dose–response equation with variable slope) and to derive contractile EC_50_ values.

## Results

### Pharmacology of Cy7-conjugated bradykinin analogs

**Fig. 1:**
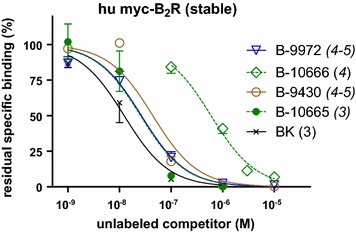
Competition of [^3^H]bradykinin (3 nM) binding to HEK 293a cells stably expressing human myc-B_2_R by bradykinin (BK) homologs. Values are the means ± SEM. of the number of duplicate determinations indicated by *n*. Average specific binding without competitor (100 %) averaged 133.8 fmol/well. IC_50_ values are reported in Table 1

A radioligand binding assay was conducted to evaluate the affinity of the novel fluorescent putative B_2_R ligands (Fig. 1, IC_50_ values and 95 % confidence limits in Table 1). Unlabeled BK itself displaced the specific binding of [^3^H]BK (3 nM) to stably expressed human recombinant B_2_Rs with a nanomolar potency. The full agonist B-9972, that incorporated several non-natural amino acid residues (Table 1), loses ~twofold affinity for the receptor, reminiscent of the order of potency at the rabbit B_2_R [2]. The Cy7 conjugate of B-9972, termed B-10666, exhibits an important ~35-fold loss of affinity vs. the parent peptide B-9972, also typical of N-prolonged agonists [8]. However, the Cy7-conjugated form of the antagonist B-9430, termed B-10665, is about equipotent to its parent peptide (Fig. 1; Table 1).

**Fig. 2:**
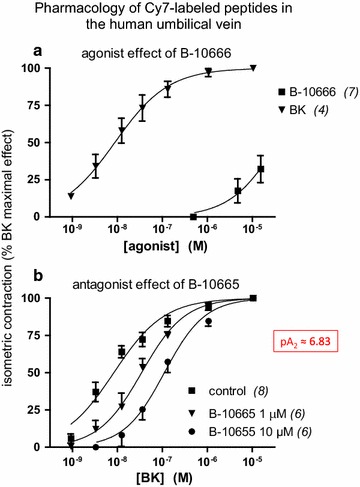
Pharmacology of the Cy7-labeled ligands in the human umbilical vein contractility assay. **a** Agonist effect of B-10666 compared to that of BK. The maximal effect mediated by the endogenous B_2_Rs have been estimated by exposing tissues to a maximal concentration of BK. **b** Competitive antagonist effect of B-10665 against BK-induced contraction. Values are mean ± SEM of the number of replicates indicated by *n*

The human umbilical vein is a contractile bioassay for the endogenous B_2_R, and was exploited to further study the pharmacology of Cy7-conjugated analogs (Fig. 2). The fluorescent peptide B-10666 was confirmed a B_2_R agonist, but with a massive 4240-fold loss of potency vs. BK based on an extrapolated concentration-effect for the former peptide (Fig. 2a). B-10665 has no direct effect on the isolated umbilical vein, but was a surmountable and rather potent antagonist of BK (Fig. 2b) with an estimated pA_2_ of 6.83, but significantly less potent than the parent peptide B-9430 in this preparation (pA_2_ 7.7) [5]. Thus, in this assay, the potency of both Cy7-conjugated peptides vs. their respective parent peptides was inferior to what could have been expected from the radioligand binding competition assay.

### Applications based on the fluorescence of Cy7-labeled B_2_R ligands: cytofluorometry

**Fig. 3:**
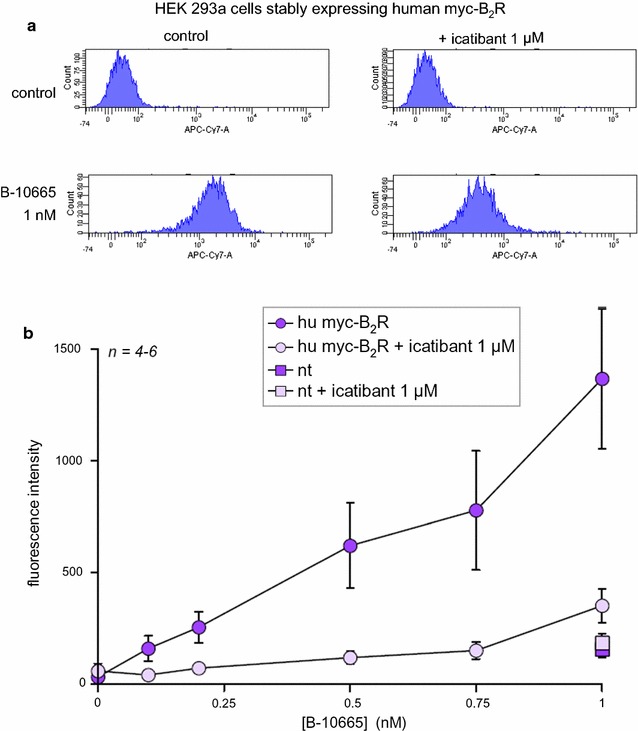
Cytofluorometric detection of B-10665 (1 nM) in detached HEK 293a cells stably expressing human myc-tagged B_2_Rs and competition of binding by the alternate antagonist icatibant. **a** Sample histograms for infrared fluorescence. Detached cells were treated for 30 min with B-10665; icatibant was applied 15 min before B-10665 when utilized. **b** Concentration-effect relationship for cell labeling with B-10665 and competition with icatibant in replicated experiments

Binding of the antagonist B-10665 was detected using its intrinsic infrared fluorescence in detached, intact HEK 293a cells that stably expressed human myc-tagged B_2_Rs (Fig. 3). The low autofluorescence of cells in the infrared range allowed detecting the binding of the probe at subnanomolar concentrations. The binding was specific as shown by its very low intensity in non-transfected cells and its extensive competition by the non-fluorescent B_2_R antagonist icatibant in co-treated cells (Fig. 3). Higher concentrations of B-10665 produced a specific binding that plateaued in the low nanomolar range, as expected with cells expressing a finite number of receptors, but non-specific fluorescence (measured in the presence of icatibant) increased considerably more, possibly related to the lipophilic nature of the fluorophore (Additional file 1: Figure S4).

### Microscopy

**Fig. 4:**
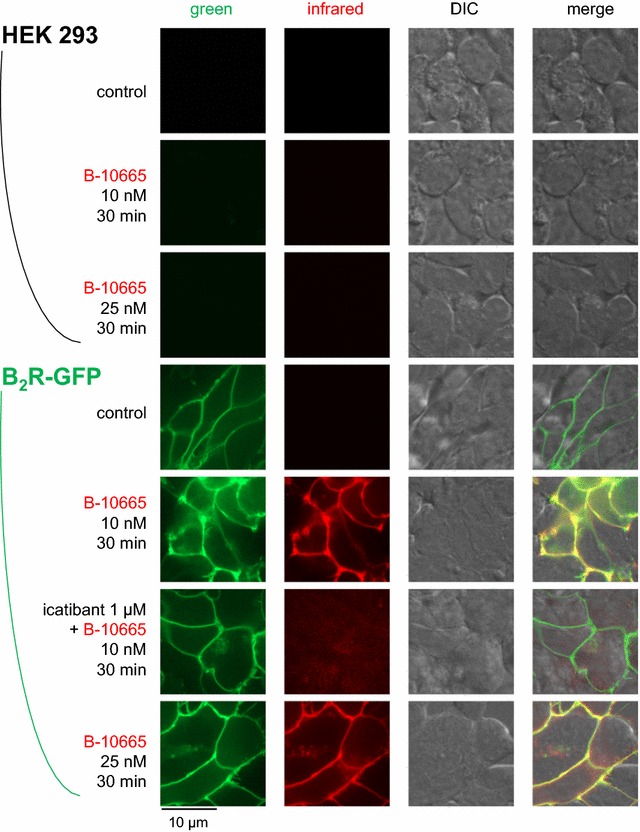
Imaging of intact HEK 293 stably expressing or not B_2_R-GFP based on the Cy7-conjugated antagonist ligand B-10665 (infrared fluorescence rendered as the *red false color*). All labelings were performed for 30 min at 37 °C, followed by rinsing. Original magnification 95×

Adherent and intact cells that stably expressed the rabbit B_2_R-GFP construction exhibited the typical membrane-associated green fluorescence corresponding to the resting receptor that was obviously absent in non-transfected HEK 293 cells (Fig. 4). There was no infrared fluorescence in resting cells, expressing or not B_2_R-GFP. The antagonist B-10665 (10 or 25 nM) labelled essentially plasma membranes in the infrared if and only if B_2_R-GFP was expressed; both signals co-localized. The plasma membrane fluorescence of B-10665 (10 nM) was prevented in B_2_R-GFP-expressing cells that had been pretreated with the B_2_R antagonist icatibant (Fig. 4), supporting the competition of the fluorescent antagonist receptor binding by the non-fluorescent antagonist.

**Fig. 5:**
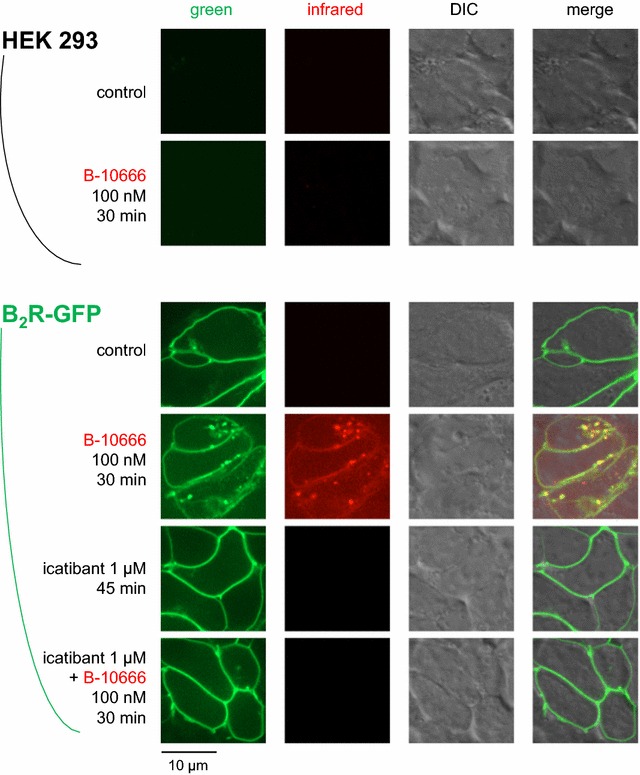
Imaging of intact HEK 293 stably expressing or not B_2_R-GFP based on the Cy7-conjugated agonist ligand B-10666 (100 nM). Presentation as in Fig. 4

The agonist B-10666 (100 nM) determined the endocytosis of a fraction of the plasma membrane receptors and colocalized, in part, with B_2_R-GFP in cells (Fig. 5). At the used concentration, the agonist did not label untransfected cells or icatibant-pretreated receptor-expressing cells, supporting a high specificity in cells that were vigorously rinsed before observation.

The comparable β_2_-adrenoceptor-GFP Topaz construction was used in additional control experiments. This fusion protein, when transiently expressed in HEK 293 cells, is mainly expressed at the plasma membrane level but with scattered intracellular labeling more intense than that of stably expressed B_2_R-GFP (Additional file 1: Figure S5). β_2_-adrenoceptor-GFP Topaz is pharmacologically reactive, as shown in isoproterenol-stimulated cells: the receptors are then translocated into endosomes that are better differentiated from the cytosolic background fluorescence at high magnification (Additional file 1: Figure S5). The agonist-induced endocytosis was prevented by a co-treatment with propranolol (Additional file 1: Figure S5), supporting the receptor identity. Both the C7-conjugated B_2_R ligands failed to label HEK 293 cells expressing β_2_-adrenoceptor-GFP Topaz (Additional file 1: Figure S6).

### Imaging of infrared fluorescence in macroscopic objects

**Fig. 6:**
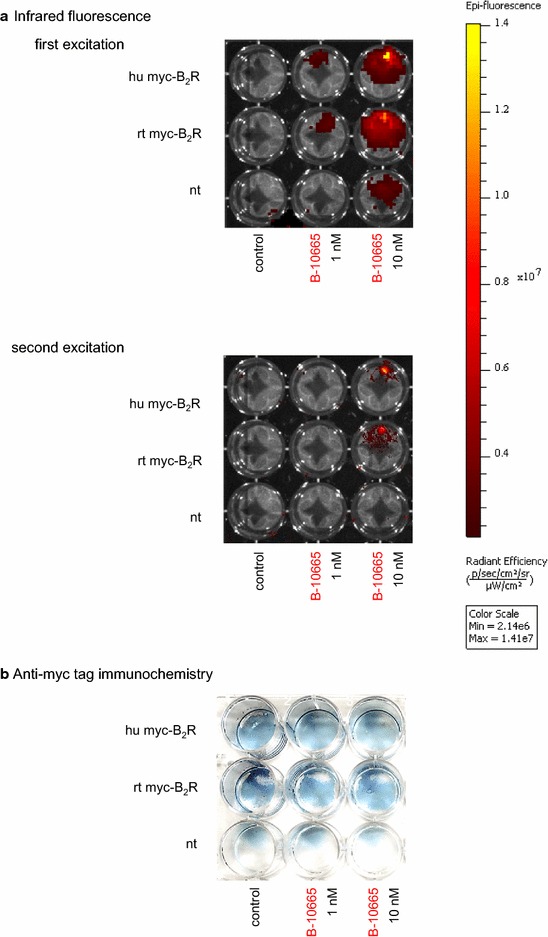
Imaging of the B_2_R distribution in a plate of wells containing HEK 293a cells that stably express myc-tagged recombinant human or rat receptors (hu myc-B_2_R, rt myc-B_2_R, respectively) or non-transfected cells (nt). **a** Infrared fluorescence following treatment with B-10665 (30 min, 37 °C, followed by ample rinsing). The *color signal* represents infrared fluorescence intensity, as quantified on the scale at the *right hand side*, and is overlayed on a *black* and *white* photograph of the object. *Top* fluorescence recorded during the first exposure (60 s); *bottom* results from the second exposure of the same object (60 s). **b** Detection of the receptors via immunohistochemistry of their myc tag (*blue precipitate*). The cell wells were submitted to the same treatments as in **a**. In both **a** and **b**, results are representative of 2 separate experiments

The fluorescence imaging system was used to probe the properties of the antagonist B-10665 in controlled conditions were cell wells containing confluent HEK 293a cells expressing human or rat B_2_Rs were photographed (Fig. 6a). B-10665 stained the cell wells (from a 24-well plate) in a concentration-dependent manner (1–10 nM), more so for the rat construction. Non-transfected cells, used as controls, showed some non-specific signal when treated with 10 nM of the probe, but not at the 1 nM concentration level. The fluorescence essentially faded after a single 60-sec acquisition period (Fig. 6a, bottom). The expression of the myc-tagged receptors was verified by an immunohistochemistry technique based on anti-myc antibodies applied to the same type of macroscopic object, a cell well plate (Fig. 6b). The blue labeling of receptor-expressing cell populations was more intense than the background staining, and the rat construction apparently more densely expressed than the human one. The pharmacological treatments had no obvious effect on the density of receptors. B-10665 (up to 250 nM) could not detect endogenous B_2_R presence in slices of human umbilical cord using this imaging system (data not shown), probably related to the low physiological density of the receptors.

## Discussion

The pair of Cy7-conjugated agonist/antagonist ligands of the B_2_R, B-10666 and B-10665, respectively, shared the same design as a previously reported pair based on the same parent peptides, fluorescein-5-thiocarbamoyl (FTC)-B-9972 and FTC-B-9430 [4]. While the conjugation to a fluorophore produced a loss of receptor affinity for both sets of peptides, the Cy7-conjugated agonist B-10666 was particularly poor, whereas FTC-B-9972 fared better (EC_50_ 108 nM in the umbilical vein contractility assay [4]). Despite this structure–activity idiosyncrasy of the agonists, B-10666 was usable in microscopy experiment where it showed the expected receptor-internalizing effect in cells incubated at 37 °C (Fig. 5). The pharmacology of the antagonist versions, B-10665 and FTC-B-9430, are very similar, with pA_2_ values of 6.83 and 6.96, respectively, against BK-induced contractions in the umbilical vein and a good receptor affinity, based on the competition of [^3^H]BK binding to recombinant receptors. Either antagonist imaged B_2_Rs at the cell surface, without endocytosis (Fig. 4) [4].

The original properties of the studied Cy7-conjugated ligands included: (1) superior performance in co-localization experiments with the B_2_R-GFP fusion protein (Figs. 4, 5), which was not possible with fluorescein-labeled ligands, supporting the very extensive co-localization of ligands with the receptor at both the cell surface and, for the agonist version, in endosomes; (2) high signal/noise ratio of nanomolar levels of B-10665 in cytofluorometry, owing to minimal cell autofluorescence in the infrared (Fig. 3); (3) detection of B_2_R populations at the macroscopic scale with essentially no autofluorescence if high densities of receptors are observed, as in cell wells expressing recombinant B_2_Rs (Fig. 6); (4) inferred resistance to peptidases, which is relevant because cell surface ectopeptidase density may vastly exceed that of BK receptors in some cells, such as human endothelial cells [14]; (5) a propensity for non-specific binding that requires careful rinsing, possibly related to the hydrophobic nature of Cy7; (6) a rapid “bleaching” observed both in microscopy and with the macroscopic imaging system (Fig. 6), but FTC-conjugated ligands do not fare better in this respect; (7) a limitation in sensitivity, common to most or all fluorophores. Thus, recombinant receptors expressed at high densities are best detected with B-10665.

## Conclusions

Despite limited sensitivity and non-specific behavior at high concentrations (Additional file 1: Figure S4), Cy7-conjugated peptidase-resistant B_2_R ligands support original imaging applications, such as co-localization with the B_2_R-GFP construction, and cytofluorometry.

## Additional file

10.1186/s13104-016-2258-1 6 Additional figures (S1–S6) and their legend.

## Abbreviations

B_2_R: B_2_ receptor
BK: bradykinin
Cy7: cyanine dye 7
DIC: differential interference contrast microscopy
GFP: green fluorescent protein
Hyp: *trans*-4-hydroxyproline
Igl: α-(2-indanyl)glycine
Oic: (3as, 7as)-octahydroindole-2-carboxylic acid
Tic: 1,2,3,4-tetrahydroisoquinoline-3-carboxylic acid
