# Supplementary material for: Infrared-emitting, peptidase-resistant fluorescent ligands of the bradykinin B2 receptor: application to cytofluorometry and imaging
Source: BMC Res Notes. 2016 Sep 26;9:452. doi: 10.1186/s13104-016-2258-1 (PMC5037861; doi:10.1186/s13104-016-2258-1)
Supplement: Supplementary file 1 — 10.1186/s13104-016-2258-1 6 Additional figures (S1–S6) and their legend. [file 13104_2016_2258_MOESM1_ESM.pdf]

Synthesis of B-10665 and B-10666

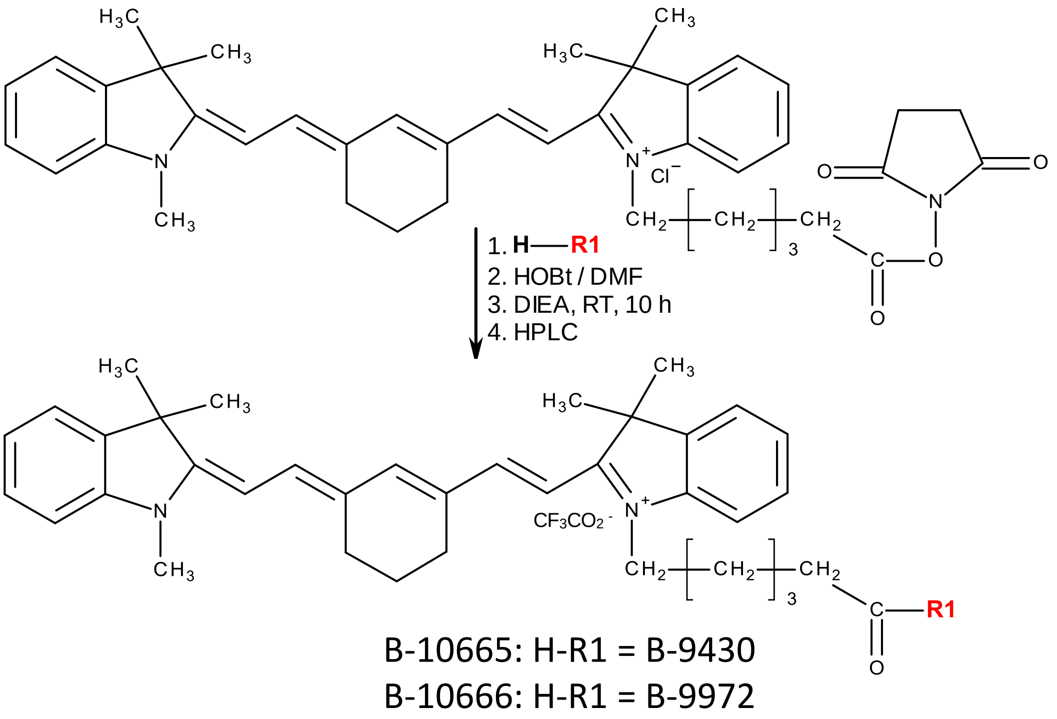

Fig. S1. Synthesis of B-10665 and B-10666. The parent peptides are B-9430 (D-Arg-[Hyp^{3},Igl^{5},D-Igl^{7},Oic^{8}]-BK) and B-9972 (D-Arg-[Hyp^{3},Igl^{5},Oic^{7},Igl^{8}]-BK), respectively.

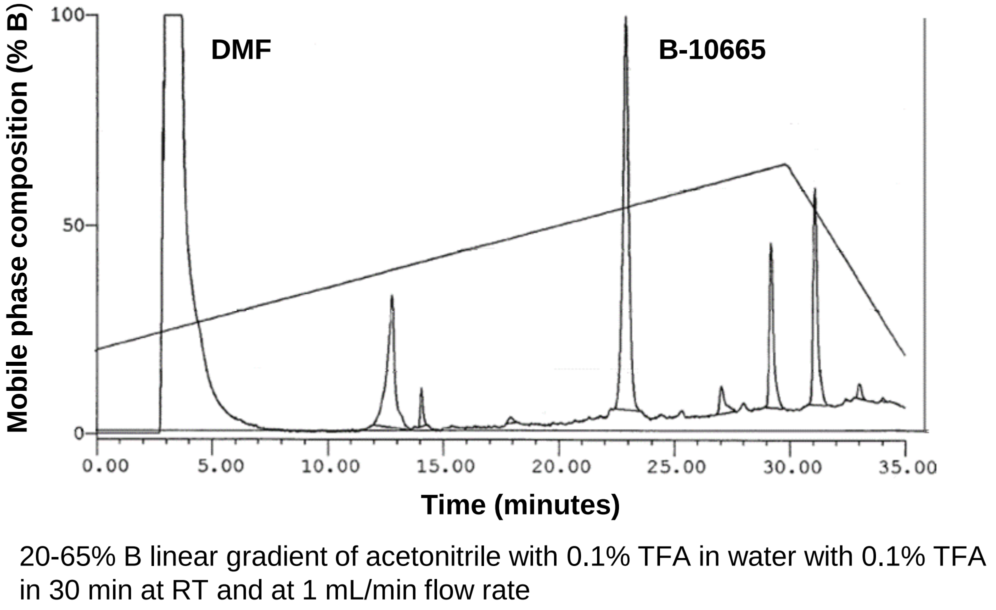

Fig. S 2. Analytical HPLC trace of the crude reaction mixture after reaction of Cy7 and B-9430 producing B-10665.

LC-MS performed on Agilent 1100 LC/Electrospray MS system

LC **B-10665**

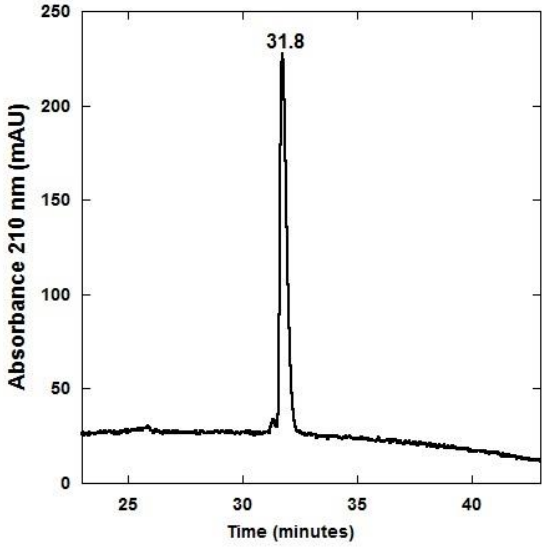

B-10666

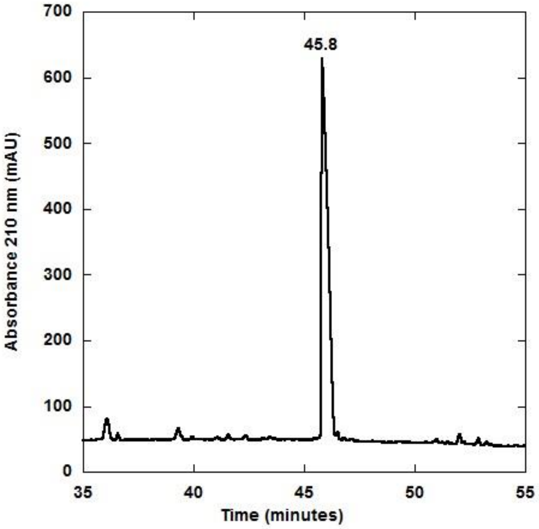

B-10665: 20-80% B linear gradient of acetonitrile with 0.1% TFA in water with 0.1% TFA at 50 ^oC, at 0.3 mL/min flow rate; gradient of 1% B/min; t_R: 31.8 min

B-10666: 2-70% B linear gradient of acetonitrile with 0.1% TFA in water with 0.1% TFA at 50 ^oC, at 0.3 mL/min flow rate; gradient of 1% B/min; t_R: 45.8 min

MS **B-10665**

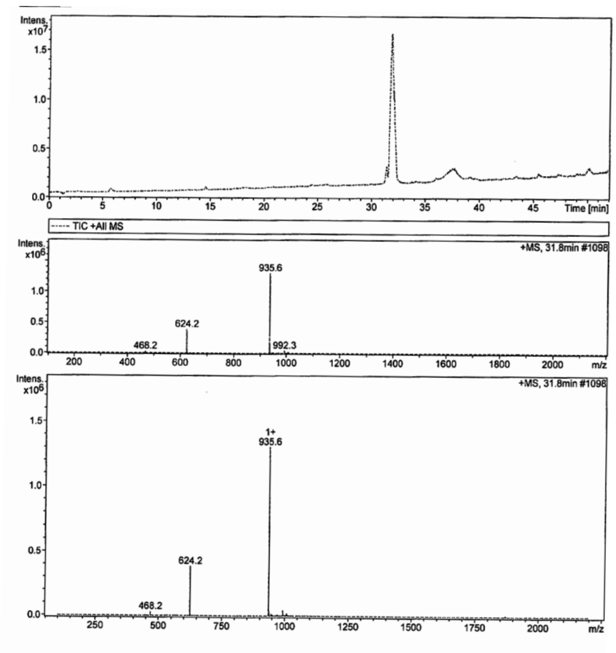

Calculated: [M+2H]+ = 936.2, found: [M+2H]+ = 935.6
Calculated: [M+3H]+ = 624.4, found: [M+3H]+ = 624.2
Calculated: [M+4H]+ = 468.6, found: [M+4H]+ = 468.2

B-10666

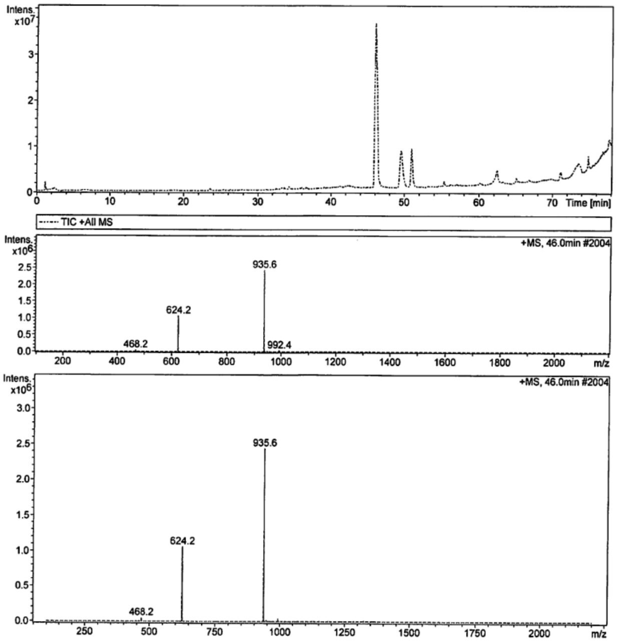

Calculated: [M+2H]+ = 936.2, found: [M+2H]+ = 935.6
Calculated: [M+3H]+ = 624.4, found: [M+3H]+ = 624.2
Calculated: [M+4H]+ = 468.6, found: [M+4H]+ = 468.2

Fig. S3. LC-MS confirmation of the identity of Cy7-conjugated B_2R ligands.

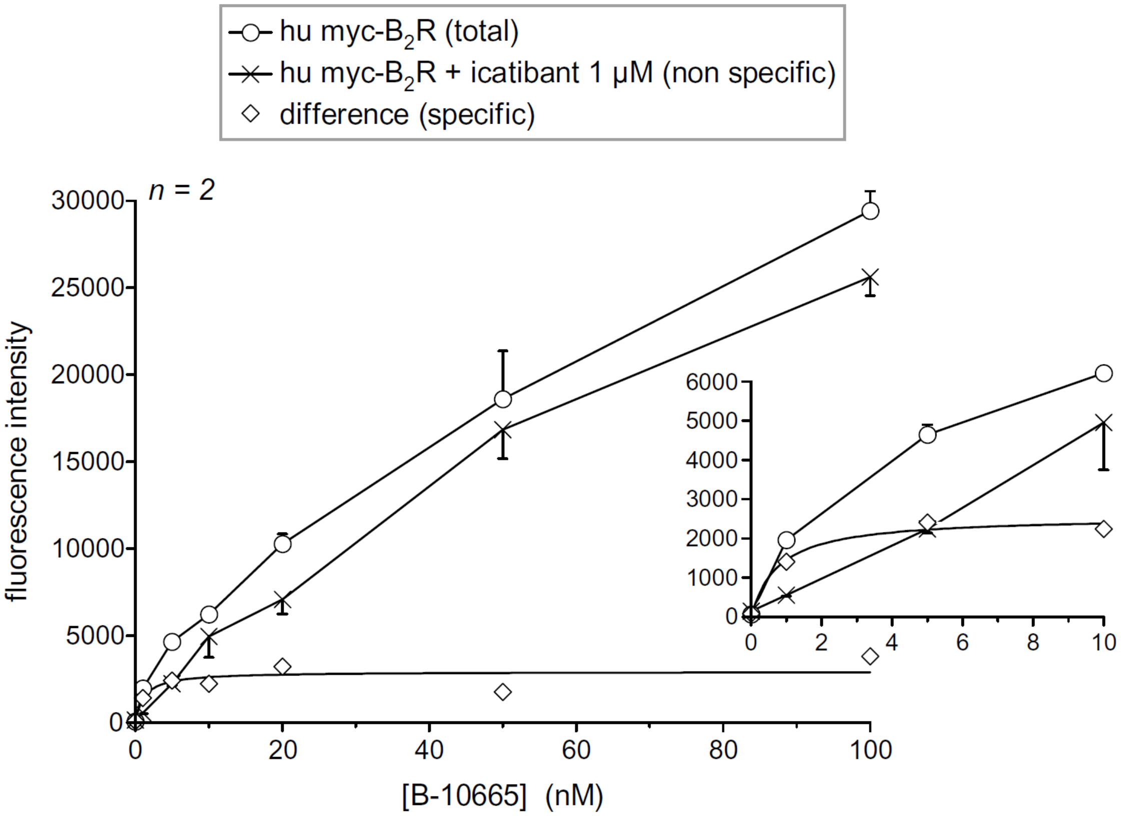

Fig. S4. Extended concentration-effect relationship for cytofluorometric determination of cell labeling with B-10665 ("total" binding") and competition with icatibant ("non-specific" binding). Detached HEK 293a cells stably expressing human myc-tagged B_2Rs were exploited (Methods as in Fig. 3). Inset: magnification of the low-concentration range of the curves. The "specific" binding, defined as the difference between the total and non-specific binding, tends to saturate, but is a small fraction of the total binding above 10 nM of B-10665.

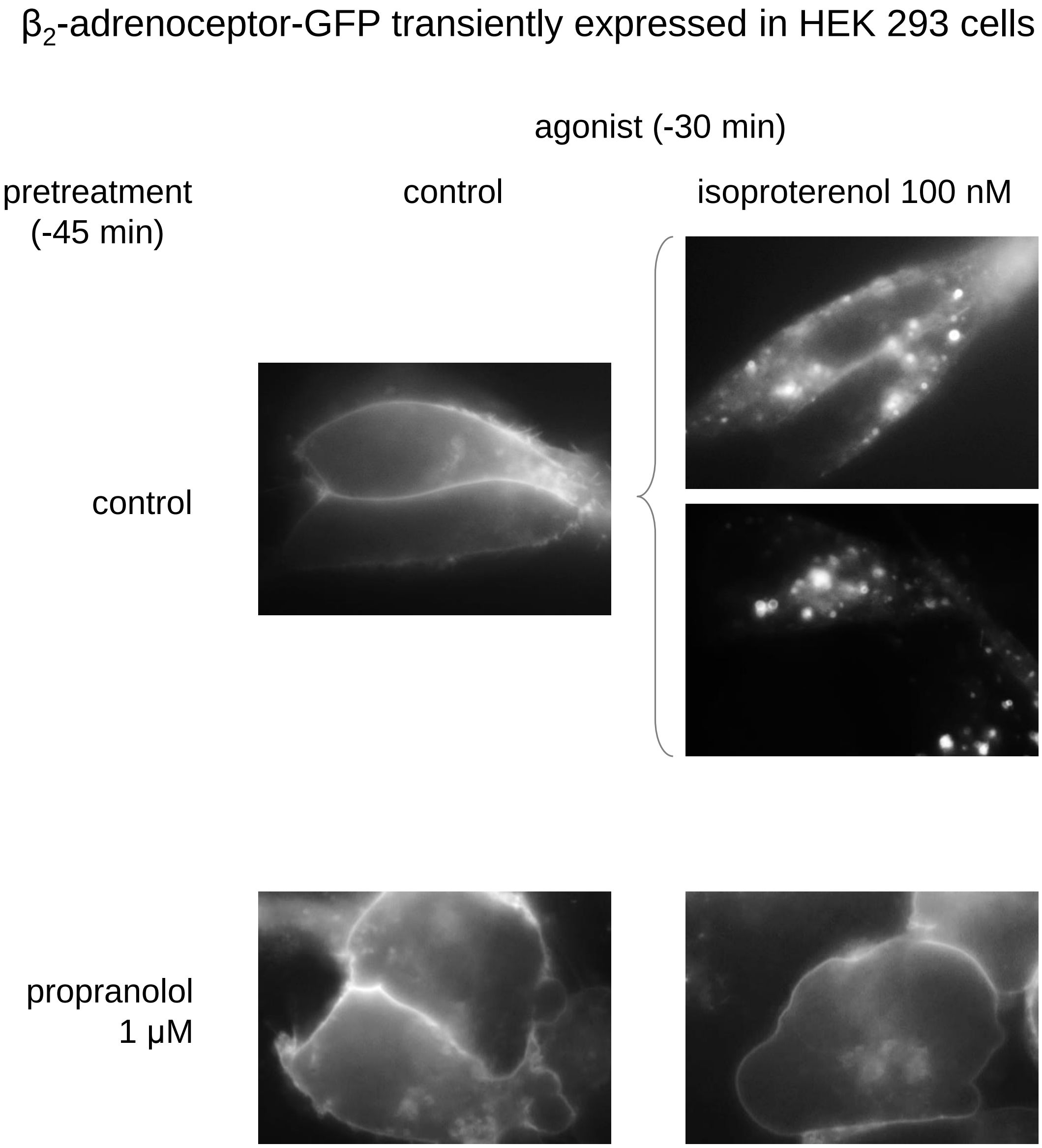

Fig. S5. Transient expression and pharmacological reactivity of the β_2-adrenoceptor-GFP Topaz fusion protein in HEK 293 cells. Cells maintained in their complete culture medium were treated as indicated at 37°C and photographed using the equipment and settings previously exploited with the BK B_2R-GFP construction in previous publications [1-3, 5]. Original magnification 1000 ×. The long sides of rectangular fields measure 55 µm.

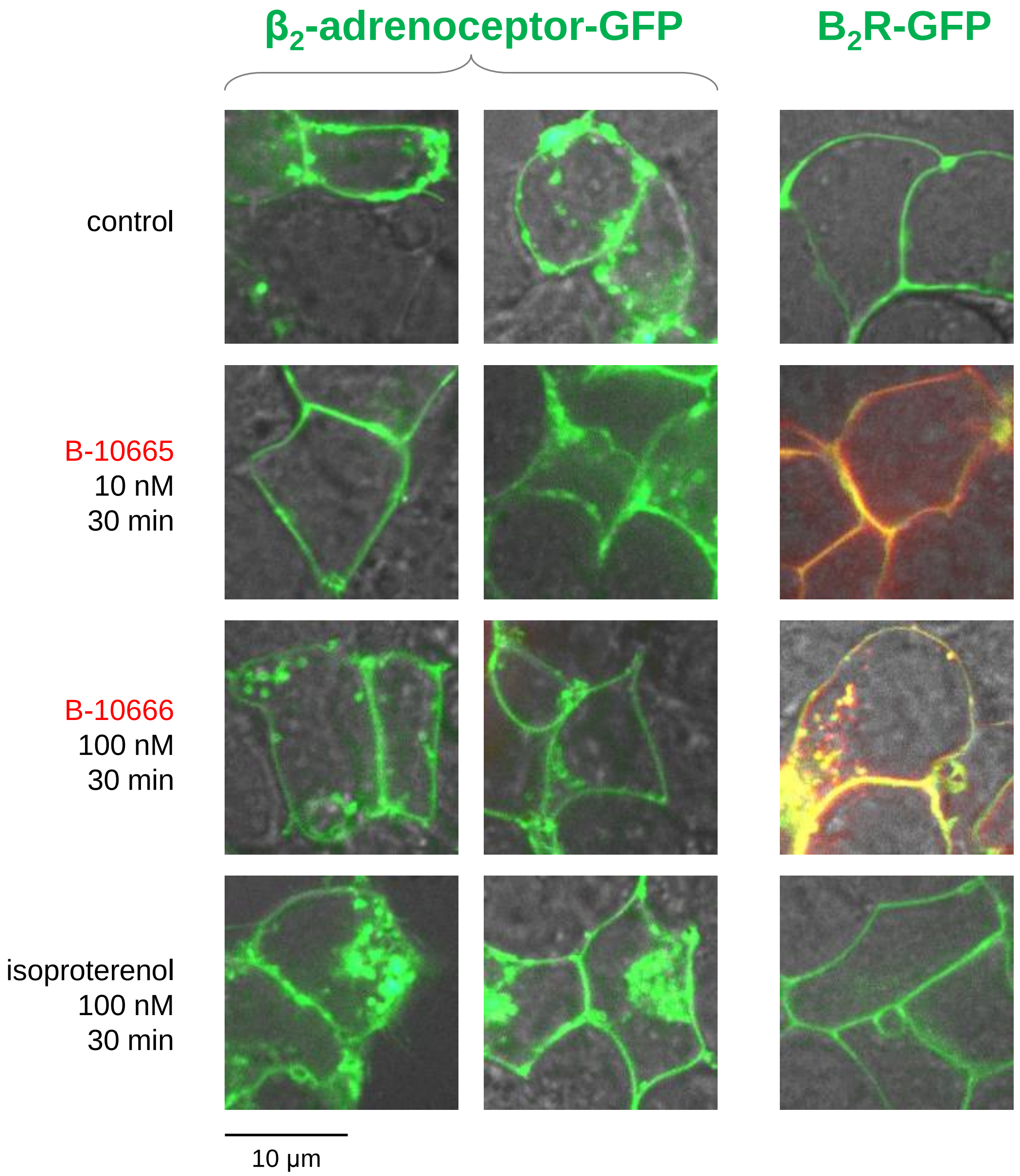

Fig. S6. Lack of labeling of cells transiently expressing the β_2-adrenoceptor-GFP Topaz fusion protein by B-10665 or B-10666. Presentation as in Fig. 4, but only the “merged” images are shown (superimposed green, infrared and DIC) . Binding of the infrared-emitting peptides (rendered as red) to cells stably expressing B_2R-GFP is shown as a positive control from the same experiment. Original magnification 95 ×.
